# Zines as artistic tools in harm reduction: bridging subjective experience and scientific knowledge

**DOI:** 10.1186/s12954-025-01216-w

**Published:** 2025-04-16

**Authors:** Marie Dos Santos, Laélia Briand-Madrid, Joachim Lévy, Jihane El Meddeb, Perrine Roux

**Affiliations:** Economic and Social Sciences, Health Systems and Medical Informatics, Marseille, France

## Abstract

Since the early days of the fight against HIV, zines have appeared to play a dual role: they encourage affected communities to voice their experiences and facilitate the exchange of information between researchers, professionals, and the individuals concerned, particularly drug users. However, few studies have examined the role of zines as a harm reduction tool within public health. This commentary draws on a two-year ethnographic study of a zine project designed for people facing drug-related challenges and vulnerabilities, such as social precarity and mental health issues. We identified various forms of engagement with the zine project, based on three key aspects. First, the zine elevates a plurality of voices, whether from the spheres of research, peer-based networks, healthcare professionals, or the streets and social precariousness. Second, the zine operates within a broader network of associations, fostering connections between local social action structures. Lastly, while the zine primarily addresses drug-related issues, it also opens space for broader topics such as precarity, gender, and migration. This thematic transversality stems from a desire to grasp the complexity of social factors that shape individual life trajectories.

The production of a zine, envisioned as a platform for expression and the promotion of an alternative subculture, promotes the participation of those most distanced from care and among the most vulnerable populations. The peer-driven collaboration throughout the zine’s creation also enables the stakeholders involved (editorial team, contributors, readers, etc.) to contribute to the emergence of diverse forms of knowledge, fostering a new understanding of reality and bridging audiences that would otherwise rarely intersect.

## Zines in the fight for people who use drugs’ rights and in the fight against HIV/AIDS

In the early 1980s, with the two political goals of autonomy for drug-using communities and the legalization of drugs, the world’s first self-support organizations and collective mobilizations - including Junky Bond and the Amsterdam-based Interest Association for Drug Users - were set up in the Netherlands. Their aim was to (i) encourage people who use drugs^1^ (PWUD) to define themselves in a way that was not solely based on pathology, and (ii) valorize the various forms of counter-culture in drug-using communities. One of the tools used to promote this mission was the world’s first fanzine (hereafter ‘zine’) written by and for PWUD, called *Spuitt 11* (the Dutch word for syringe). Launched in 1981 *Spuitt 11* was developed to give a voice to PWUD, and to facilitate knowledge sharing in a language and format that was accessible and appealing to the people concerned. The subsequent years saw the launch of several other zines for drug-using communities in different countries around the world. To mention the main zines published in English, in Australia, three different zines written for and designed by people who use drugs and for people who use drugs were edited. The first one was WHACK magazine is an ongoing publication of Harm Reduction Victoria (HRVic) which was first published in July 1988 and continues to be published in 2025; then Junkmail was first published in 1991 through to 2010 by AIVL (which stands for the Australian Injecting & Illicit Drug Users League); finally, Users News’ which started as ‘NUAA News’ and subsequently changed its name was first published in 1991, published 99 editions with the last edition published in 2022. The first editor of NUAA News was Chris Jones, a poet, artist and editor, and a vocal Sydney activist for gay rights and injecting drug users. In New Zealand, NZ NEP has had a strong history of regional/ city and national zines from their earliest days in mid-1980s, underpinned by a peer-led approach. ‘On Board’ was the national zine and ‘Mainline’ was a Christchurch zine and other zines provided harm reduction education, peer stories, and local creativity through images, cartoons and the like. Innovations such as CDs of local music was a feature of a vibrant, community-oriented outlook that worked to empower and advocate for drug users particularly people who injected drugs. Finally in UK, *Black Poppy Publications* (1998) was edited in partnership with Exchange Supplies.

Some English-language zines have certainly been overlooked here. This commentary does not claim to be a comprehensive review of all drug user zines that may have been produced over the past decades, but rather draws on the limited information available to contribute the existing discussion on this important area of harm reduction practice^2^.

Right from the outset of the HIV epidemic at the beginning of the 1980s, zines for PWUD were active in the fight against the virus. They incorporated articles on PWUDs’ experiential knowledge of drug use and sexuality, as well as articles on research-based knowledge about modes of transmission with a view to familiarizing people affected by HIV with biomedical knowledge, so that they could present themselves as legitimate actors in their struggle to negotiate with health professionals and public authorities on issues of access to care, treatment (e.g., community involvement in clinical trials), and harm reduction (Epstein 1995).

This objective is reflected in ‘The Denver Principles’; published in a manifesto in 1983, and based on the tenet ‘nothing about us without us’, these principles encouraged people concerned by HIV to participate in public debate and speak out in the media on HIV issues in order to be heard by civil society, care providers and politicians.

In the forty years since the first zines for PWUD were published, a small number have taken inspiration from punk and do-it-yourself cultures (Brouwer, 1992) to develop radical content on HIV, drug use and sexuality, where counter discourse opposes the dominant discourse. For example, *Diseased Pariah News* and *Infected Faggot Perspectives* - published from 1980 to 1999 - aimed to offer alternative content to what was being published in the mass media on the subject of HIV/AIDS. The former focused more on the experience of HIV-positive people, while the latter offered political content around Queer activism. According to Brouwer, the objective of these two zines was to constitute “counterpublics”, or to cite Fraser (1992), “parallel discursive arenas”.

For the artist activist John Douglas Crimp, as a member of Act Up-New York, zines and cultural practices actively contributed to the fight against AIDS and its cultural consequences (Crimp, 2016). He participated to Queer theory, inspired by Foucault, in order to re-think the AIDS crisis by taking into account the forms of normativity imposed and by acting on sexualities and social practices (as drugs uses). He, like others, perceived that artistic practices were capable of reflecting individuals’ sensitivity and emotions in the context of AIDS beyond mere medical considerations.

Another example is the Queer and feminist American zine *Junkphood* which spread information on harm reduction and injection practices. Wieloch shows how *Junkphood* contributed to the creation of a collective identity among PWUD, and to the formation of a new social movement on the margins of conventional society whose culture existed outside established institutions [15]. By valorizing drug use with statements like “I am very proud to be a junkie”, the stated objectives of the zine were to reverse the stigma surrounding drug use, to encourage readers to self-define themselves positively, and to rebuke their “spoiled identity” [7]. However, *Junkphood* also offered advice and provided health information. For example, it published articles on the effects of drugs, the symptoms of the different types of hepatitis, and on safe injection techniques.

Examples of currently active French-language PWUD zines are *Asud Magazine* in France and *L’injecteur* in Quebec, Canada. The former is the foremost publication in France by and for PWUD. First published in 1992 by the association of the same name [3], *Asud Magazine* was originally designed as a medium to inform and to raise awareness, its mission being to have the French’s 1970 law on the prohibition of drugs changed. Today, through its zine and activism, the association continues to tackle political and public health issues concerning PWUD.

*L’injecteur* is published by the Association des intervenants en dépendance du Québec (loosely translated as the Quebec Association of Addiction Stakeholders). First published in 2005 by “people using drugs and for people using drugs” [2], *L’injecteur* was created to share drug-use stakeholders’ experiences, testimonies and harm reduction strategies. Today, prints from *L’injecteur* and elements of the zine’s style and identity are used in various prevention campaigns for PWUD by various organizations in Quebec.

*L’injecteur* provided the inspiration for the development of *Sang d’Encre*, the French zine project which we will discuss in the present article.

## Harm reduction epistemologies from a social sciences perspective

The relative lack of research on zines in the area of harm reduction reflects how evaluation studies of social support tools do not take into account the fact that experiential knowledge produces legitimate forms of knowledge– in particular artistic-based and emotional-based knowledge– which can be help guide healthcare policy. More generally, experiential knowledge cannot always be expressed in standard scientific textual format. Expressing experiential knowledge in more artistic forms fosters the production of emotion-based knowledge. In the field of harm reduction, taking emotion-based knowledge about drug use and sexuality into account has helped to shape political landscapes and has played a role in defining the issues related to the fight against HIV [8]. However, incorporating experiential knowledge in social support research has a cost in terms of resources and the choice not to investigate it reflects the neoliberal logic that health costs should always be recorded in an accounting register [5]. The consequence is that PWUD’s experiential knowledge is mostly discredited in scientific and political debates surrounding drug use and addiction, a field where the biomedical approach continues to dominate [13].

Anchored in a social perspective of public health, harm reduction is an evidence-based, person-centered approach to minimize the negative health, social and legal impacts associated with drug use. It is based on knowledge of epidemiological and medical sciences and the experiential knowledge of the people concerned (Kippax and Race 2003). Harm reduction studies suggest that the promotion of experiential knowledge, in all its diversity, is essential to improve PWUD’s health, well-being and quality of life. Although empowerment is a core objective of harm reduction, its institutionalization over the past decade has at times paradoxically led to the exclusion of PWUD, thereby hindering their self-governance and the way they produce knowledge about themselves [9].

An intersectional approach to research makes it possible to understand the way in which different forms of structural domination - in other words, different forms of privilege or oppression - contribute to discredit PWUD’s experiential knowledge. These forms of “epistemic injustice” [4, 6] have consequences on drug use and can increase related risks through misinformation (Collins et al. 2019). In this sense, the development of harm reduction can be understood as a reaction to the different forms of structural domination that weigh on PWUD [15]; Bourgois and Schonberg 2009, Smith, 2012). In more concrete terms, harm reduction aims to shift the dominant discourse surrounding addiction - which places all responsibility squarely on the individual’s shoulders - to one where societal causes are taken into account, with a view to better understanding the context and the social inequalities in which risk-taking takes place.

Ethnographers conducting fieldwork claim to have expert knowledge about drugs; however, this is different from PWUD’s experiential knowledge. Several studies attest to the ability of social researchers to provide a detailed understanding of social and health issues related to drug use (Lindesmith 1947; Becker 1966; Preble and Casey 1969). Ethnography serves as both an instrument and effect of governance in the harm reduction field and in fight against HIV (Campbell and Shaw 2008). The positions and roles of social science researchers (ethnographers, sociologists, etc.) in meeting places where PWUD rights are discussed and harm reduction norms produced are negotiated with drug use stakeholders. These meetings contribute to reciprocal acculturation between both groups. For researchers, the scientific effort to distance oneself from emotions is counterbalanced by the challenges of co-production and each individual’s subjectivity and sensitivity.

Anthropologists are experts in what happens on the ground; they help to increase PWUD’s awareness of harm reduction. Together with social science researchers and medical researchers, anthropologists and PWUD become co-producers (i) of knowledge about harm reduction, (ii) of the discourse surrounding harm reduction as a field of research, and (iii) of new harm reduction norms (Walker 2021). Specifically, PWUD - who are the *insiders* - produce knowledge through the sharing of experiences and testimonies; often this knowledge is non-verbal, artistic-based and is disseminated through cultural productions (paintings, literature, cinema, theatre etc.). These forms of experience-based knowledge production encourage the decompartmentalization of categories, and a rethinking of issues of health and drug use by restoring them to their cultural context of production. As suggested by Ingrid Walker, instead of silencing drug use, the construction and disclosure of individual and collective discourses around this topic leads to better epistemic justice, as all too often, research focuses only on problematic uses, thereby hampering any understanding of the extent and diversity of drug use practices (Walker 2021).

## Key findings from the *SaNg d’encre zine* project

The remainder of the commentary will specifically focus on the *SaNg d’EnCRe zine* project, which illustrates the extent to which zines are essential to the harm reduction, at various levels.

### At the academic level

Fieldnotes 1st author.

*“Researchers? Have you ever met a researcher?” a peer health mediator asks another member of SaNg d’EnCRe mockingly*,* as I [a researcher] wait with the latter for the steering committee to commence.*

The *SaNg d’EnCRe zine* project is a partnership between the French PWUD organization Nouvelle Aube which supports people living in very precarious situations (living in squats, drug use-related problems, etc.), and the community-based participatory research (CBPR) team SanteRcom, which is part of the French public health research unit Sesstim. Both organizations are located in Marseille. The project was conceived in 2018 after the two organizations worked together to share the results of a previous CBPR study on injectable buprenorphine they had collaborated on [12] by publishing a booklet containing a summary of the CBPR study results, related testimonials and participants’ illustrations [11].

As well as co-producing the zine and its content, the SanteRcom research team also performs participant observation of the project in order to document the Zine’s publishing process. In this context, the team conducted a socio-anthropological study between January 2020 and August 2022. The three researchers involved in the study (authors of this article) actively participated in the zine’s various steering committee meetings. These meetings were designed to be spaces where PWUD stakeholders could become more aware of the world of science and where researchers could increase their understanding of PWUD’s real-life harm reduction practices and their reasoning for these practices within a perspective of generating more relevant research questions and of combating researcher ignorance, which is often related to stigma.

### At the community level: contributing to harm reduction through the production of different forms of knowledge

In terms of the members of Nouvelle Aube responsible for the production of the *SaNg d’EnCRe* zine, two are paid employees while the others are service providers (e.g., graphic designer, workshop organizers) or volunteers. There is a close affinity between the zine’s different team members, and most are part of the historical core of the Nouvelle Aube association. The zine’s production process covers a wide variety of tasks and roles from participation in steering committees, to the production of zine issues, to proofreading, to text production, and to distribution. This allocation of roles constantly evolves through a process of continuous learning.

*“So we wanted it to be accessible*,* we also wanted these people from the street to be able to express themselves as well.” (Interview*,* man*,* historical core*,* employee)*.

The zine’s goal is to give a voice to marginalized populations and to promote health through content tailored to the targeted readership. The steering committee meetings aim to ensure balanced content which reflects the team’s heterogeneous composition of artists, writers, public health researchers, social workers, sociologists, and other stakeholders. The diversity of text formats is a key element, as the zine aims to cater for different levels of reading (e.g., skimming, scanning, detailed). Heated negotiations are not uncommon. PWUD’s contributions are, for the most part, left unedited (apart from spelling corrections, etc.) to ensure that their choice of discourse themes, the tone of their contribution, and the emotional weight of the latter are all respected. For many contributors, participating in the zine’s content is much more than simply sending in a text or a drawing; it provides them with the opportunity to reflect on their own experiences of marginalization.

Fieldnotes 1st author.

*A testimony was read out during the Steering Committee meeting; we were very moved. It was a short text*,* written in the first person*,* by a woman who talked about having suffered incest. It was right in the middle of #MetooIncest. The author probably seized on this opportunity to speak freely in order to finally open up*,* to get this experience out of her. During our collective discussion to define what would appear in the next issue*,* we mentioned the violence of the text*,* and just like we do every time we receive a testimony*,* we thought about including it in a theme-based section. That’s how Dorothée Dussy*,* an anthropologist who has worked on incest (Dussy 2021) was contacted. She wrote an article advocating the recognition of the rights of victims and a real policy on the fight against intra-family violence. A harm reduction team working in the party drug scene was also approached for the same issue to present their new strategy to prevent gender-based and sexual violence*,* and the tools they are developing.*

Through the *SaNg d’EnCRe* zine, Nouvelle Aube and Santercom jointly strive to provide a space that allows intimate experiences to be shared, and to offer keys to understanding the structural issues that weigh on individual trajectories - in particular on people’s behavior in the face of risks - through reflexive and social science-based contributions. In this sense, text-based testimonies which recount life journeys in all their complexity and which highlight different forms of oppression are extremely useful both for analyzing the intersection of different forms of structural domination, and for reflecting on possible actions to fight injustice.

### At the local level: a ‘hand-to-hand’ zine

*“It often happens that I walk around with copies of SaNg d’EnCRe*,* and when I talk to people I can give them the zine. It’s a great support to explain outreach*,* it’s a bit like a work tool.” (Interview*,* man*,* historical core*,* employee)*.

In a context where digital technology is increasingly present, and where there is growing interest by public health actors in the use of smartphone applications for harm reduction, the publication of a paper-based zine like *SaNg d’EnCRe*– produced during workshops in a PWUD association’s (Nouvelle Aube) premises, and distributed hand to hand by the zine’s partners– constitutes an outreach activity that reconnects with the most traditional of harm reduction approaches, specifically getting as close as possible to target audiences (O’Hare 2007). During the COVID-19 pandemic, it was decided to continue distributing the zine in paper format to ensure that marginalized target audiences would be reached [1, 10]. Moreover, the team behind the zine have decided to keep the zine’s original format, which is reminiscent of paper-based counter-culture punk fanzines from the 1990s, in order to foster peer identification of this counter-culture genre while prioritizing content that focuses more on health issues. Distributed directly to people living in the street and other populations experiencing significant social precarity who are targeted by frontline outreach actions, the material nature of this paper-based zine represents a kind of symbolic (rather than instrumental) donation. Attachment to this type of symbolic donation does not create a sense of dependency or debt; rather, it strengthens a sense of belonging, and fosters the creation of new connections between people.

### At the national level: zines as a social transformation tool?

*“We do things; I don’t want to show off here*,* but we manage to do super cool things with them [i.e.*,* the zine’s contributors]” (Interview*,* man*,* historical core*,* service provider)*.

Zines such as *SaNg d’EnCRe*, which are conceived as a space for the expression and promotion of an alternative sub-culture, and which are distributed locally, have very little visibility in terms of public authorities and the uninitiated public. This lack of external visibility can lead to zines being seen as a safe space where experiences can be freely expressed. This is especially important in the current context where creating common knowledge about drugs seems increasingly difficult because of various - and sometimes aggressive - forms of rejection and stigmatization of PWUD, where they are labeled as either irresponsible patients or dangerous offenders. Many zines are only published over a relatively short period of time; in a way, this strengthens their agency, as it constitutes their alternative essence. The rebellious dimension of zines enables PWUD to reconnect with the political aspect of harm reduction. They operate within the margins of society to redefine, generate, and adjust norms in response to contingencies. In this way, zines contribute to the gradual and successive development of policies related to harm reduction and health promotion.

## Conclusion

By discussing, evaluating, and including testimonials and other contributions by drug use stakeholders in every issue, the production process behind the *SaNg d’EnCRe* zine validates the importance of experiential knowledge as a key element in harm reduction. Zines as a harm reduction tool offer a space for the production of specific knowledge, lying at the crossroads between experiential, professional, and scientific knowledge. As cultural artifacts, they contribute to the creation of “politics of emotions” [8, 14] that help shape an action framework, where the expression of feelings finds a place and a means to emerge.

Participation in the production of a harm reduction zine requires organization and compromise. Public policies must provide a set of hetero-supports through strong financial support in order to help the people involved in the production process to increase their autonomy. Finally, as effective harm reduction tools, zines can be considered a valuable means for sharing both theoretical and experiential knowledge in the wider context of public health.

## Data Availability

No datasets were generated or analysed during the current study.

## Footnotes

1 In this article, we have chosen to follow the recommendations of the International Network of People who Use Drugs (INPUD) by using the terms ‘people who use dugs’ and ‘people who inject drugs’, as they are considered less stigmatizing than the term ‘drug users’.

2 I’d like to thank Annie Madden, editor of Harm reduction Journal, for her insights into the English-language zines mentioned here.
